# Geographic variation and neighbourhood correlates of mental health-related hospitalizations and emergency department visits in children and youth during the COVID-19 pandemic: a population-based study

**DOI:** 10.3389/frcha.2026.1575531

**Published:** 2026-05-18

**Authors:** Tony Antoniou, Peter Gozdyra, Daniel Fridman, Kathleen Pajer, Tara Gomes, Melanie Penner, Mina Tadrous, David N. Juurlink, William Gardner

**Affiliations:** 1 Li Ka Shing Knowledge Institute, St. Michael’s Hospital, Toronto, ON, Canada; 2 ICES, Toronto, ON, Canada; 3 Department of Family and Community Medicine, University of Toronto, Toronto, ON, Canada; 4 Department of Family and Community Medicine, St. Michael’s Hospital, Toronto, ON, Canada; 5 Children’s Hospital of Eastern Ontario Research Institute, Ottawa, ON, Canada; 6 Department of Psychiatry, University of Ottawa, Ottawa, ON, Canada; 7 Leslie Dan Faculty of Pharmacy, University of Toronto, Toronto, ON, Canada; 8 Institute of Health Policy, Management, and Evaluation, University of Toronto, Toronto, ON, Canada; 9 Autism Research Centre, Bloorview Research Institute, Holland Bloorview Kids Rehabilitation Hospital, Toronto, ON, Canada; 10 Department of Pediatrics, University of Toronto, Toronto, ON, Canada; 11 Department of Medicine, University of Toronto, Toronto, ON, Canada; 12 School of Epidemiology and Public Health, University of Ottawa, Ottawa, ON, Canada; 13 ICES, Ottawa, ON, Canada

**Keywords:** adolescent, child, COVID-19, emergency department (ED) utilization, hospitalization, mental health services, spatial analysis, young adult

## Abstract

**Background:**

The COVID-19 pandemic was associated with increased mental health-related hospitalizations and emergency department (ED) visits in children and youth. However, population-based studies examining geographic variation in, and neighbourhood correlates of, acute mental health service use in this population during the pandemic are lacking.

**Methods:**

We conducted a population-based study examining geographic variation in and neighbourhood correlates of mental health-related hospitalizations and ED visits among Ontario children and youth. We used hot spot analyses to visualize geographic variation in service use before (March 2017 to February 2020) and during (March 2020 to July 2021) the pandemic. We used spatial regression models to estimate unstandardized and standardized coefficients of the association between neighbourhood characteristics and acute mental health service use.

**Results:**

Mental health-related hospitalization and ED visit rates were highest in rural and northern Ontario. The variables most strongly associated with hospitalization rates within the same neighbourhood were neighbourhood income and family size. A one standard deviation (SD) increase in income was associated with a 0.50 (95% CI: −0.56 to −0.43) SD decrease in hospitalization rates, while a one SD increase in family size was associated with a 0.35 (95% CI: 0.29–0.42) SD increase in hospitalization rates. Additionally, increases of one SD in neighbourhood income, the proportion of the population speaking neither English nor French, and remoteness were associated with respective increases in the SD of hospitalization rates of in adjacent areas. Neighbourhood income and remoteness were strongly associated with ED visit rates, with each standard deviation increase in income associated with a 0.52 (95% CI: −0.57 to −0.47) standard deviation decrease in ED visits while each standard deviation increase in remoteness was associated with a 0.29 (95% CI: 0.21–0.37) standard deviation increase in ED visit rates.

**Conclusion:**

Geographic location and neighbourhood socioeconomic and demographic factors influenced acute mental health service use among children and youth during the pandemic. These findings highlight the need for targeted social interventions that address neighbourhood disparities and bolster mental health services in vulnerable regions, particularly in preparation for future large-scale disruptions.

## Introduction

COVID-19-associated school closures and other public health measures were associated with an increased prevalence of depression, anxiety and behavioural problems in children and youth (1–3). These changes were accompanied by a corresponding increase in acute mental health service use. Studies from multiple regions have described increased rates of mental health-related emergency department (ED) visits and hospitalizations among children and youth during the pandemic (4–15). These studies have generally demonstrated increases in emergency department visits and hospitalizations for anxiety, depression, eating disorders and self-harm among children and youth, particularly among females and older adolescents (15). The reasons underlying the deterioration in mental health among children and youth during the pandemic are multifactorial and include reduced physical activity, increased social isolation, home confinement and increased screen time (16–18). Family-level stressors, such as parental depression and anxiety, increased family violence, and caregiver loss, have also been identified as contributing factors (19–23).

However, most research examining correlates of the mental health of children and youth during the pandemic has focused on individual and familial determinants, rather than on broader contextual factors influencing acute mental health service use (24, 25). This is an important gap for several reasons. First, pre-pandemic studies have shown that living in neighbourhoods characterized by lower socioeconomic status (26, 27), lower levels of social cohesion (28–31), and lower levels of safety (e.g., crime, perceived neighbourhood safety) (32–36) is associated with poor mental health outcomes in children and youth. Moreover, these neighbourhood characteristics influence mental health independent of individual socioeconomic deprivation and family-level stressors (26). Neighbourhood socioeconomic disadvantage is a well-documented risk factor for child and youth mental health problems. Specifically, a United States (US) multilevel, longitudinal study of 2,805 children between the ages of 5 and 11 found socioeconomic (SES) gradients in pediatric mental health, with the prevalence of internalizing problems (i.e., depression, anxiety, withdrawal) being 21.5%, 18.3% and 11.5% among children in low, medium and high SES neighbourhoods, respectively (26). The neighbourhood effect persisted even after accounting for family demographic characteristics, maternal depression, and previous mental health scores. Moreover, 11.1% of the variance in internalizing symptom scores was attributable to between-neighbourhood differences, highlighting the role of neighbourhood context independent of individual and family characteristics (26). Notably, neighbourhood collective efficacy and organizational participation were associated with better mental health outcomes. The relationship between social cohesion and mental health of children and youth has been further established in other studies. Specifically, in a longitudinal study of 2,385 children between the ages of 3 and 15 years, severe neighbourhood economic disadvantage was associated with high initial levels of both internalizing and externalizing symptoms in early childhood after controlling for individual and family-level factors (31). However, children living in neighbourhoods with a higher level of social cohesion had lower levels of both internalizing and externalizing symptoms (31).

Neighbourhood safety, encompassing both exposure to crime and perceived danger, is another neighbourhood-level determinant of mental health in children and youth. A meta-analysis of 114 studies found that community violence was particularly associated with posttraumatic stress disorder (PTSD) and externalizing problems (32). Although victimization was the strongest predictor of mental health conditions, PTSD was also associated with witnessing or hearing about violence. The effects of community violence varied by age. Stronger associations with externalizing behaviours were observed in adolescents, while relatively greater vulnerability to internalizing symptoms was observed in younger children (32). A similar finding among adolescents was observed in a longitudinal study of 364 low-income urban youth followed annually for three years, with exposure to community violence more strongly associated with externalizing relative to internalizing symptoms (34). In a study of 3,291 ten-year old children, direct experiences of harm, parental safety perceptions and community crime statistics were each independently associated with internalizing symptoms (33). Furthermore, direct harm experiences and parent's safety perceptions were also associated with externalizing behaviours (33).

Features of the neighbourhood-built environment have also been associated with the mental health of children and youth. Systematic reviews of exposure to nature and green space have found beneficial associations with reduced stress, positive mood, emotional well-being, overall mental health, and improvements in attention deficit hyperactivity disorder (ADHD) symptoms, resilience and health-related quality of life (37–39). Although some studies have found improvements in depressive symptoms, the evidence for this outcome is mixed and more limited. A recent systematic review of the broader built environment found that neighbourhood green and blue spaces were positively related to health-related quality of life in school aged children, although associations with other neighbourhood features such as street connectivity and playground density were less consistent (40).

Housing is another feature of the neighbourhood-built environment influencing the mental health of children and youth. In a US prospective cohort of 1,339 participants, childhood housing insecurity, defined as reduced standards of living, frequent residential moves, foster care status and involuntary separation from home, was associated with higher anxiety and depression symptom scores during childhood (41). Furthermore, childhood housing insecurity predicted higher depression symptom scores in adulthood after adjustment for childhood poverty and other confounders (41). In another study using data from the 2022 National Survey of Children's Health, children experiencing housing instability, defined as concern about eviction, multiple residential moves and being behind on rent or mortgage payments, were more likely to have anxiety and depression after accounting for sociodemographic variables (42). However, children with depression or anxiety and housing instability were 38% to 39% less likely to receive mental health care than stably housed children (42). Moreover, children with depression were 22 times more likely to receive mental health treatment if they had stable housing, whereas children with anxiety were 17 times more likely to receive treatment if they had stable housing.

Second, studies examining the influence of neighbourhood on the mental health of children and youth have generally not been population-based, have mostly relied on self-reported mental health symptom ascertainment, and have not examined acute mental health service use (25–36). Third, mental health-related hospitalizations and emergency department visits are markers of disease severity that may reflect geographic disparities in mental health care access and neighbourhood socioeconomic status (41, 42). Population-based studies examining geographic variation in, and neighbourhood correlates of, acute mental health service use during the COVID-19 pandemic are therefore important for policy and practice. Data generated from such research can inform proactive measures to mitigate the impact of future large-scale disruptions and guide the rapid deployment of scalable mental health interventions in vulnerable communities. Accordingly, we examined neighbourhood correlates of mental health-related hospitalizations and ED visits during the COVID-19 pandemic among the entire population of individuals aged 0 to 24 years in Ontario, home to approximately 40% of Canadian children and youth (43). Guided by prior research, we analyzed neighbourhood characteristics associated with the mental health of children and youth, including neighbourhood socioeconomic status (median neighbourhood income), housing composition and social support structures (family size, multi-family households) and quality of the physical environment (walkability). We further included variables we postulated could influence access to mental health services, including remoteness and linguistic or cultural barriers to care (proportion of the population that are recent immigrants, visible minorities, or without knowledge of English or French). Finally, we included variables reflecting pandemic-related stress and infection exposure risk, including neighbourhood burden of COVID-19 and the proportion of residents employed in essential services.

We specifically addressed three research questions in this study. First, what was the geographic variation in mental health-related hospitalizations and emergency department visits among children and youth during the COVID-19 pandemic in Ontario? Second, which neighbourhood characteristics were associated with acute mental health service use within the same neighbourhood during the pandemic? Third, did neighbourhood characteristics exhibit spillover effects, where characteristics of one neighbourhood may influence mental health service use in adjacent neighbourhoods. We hypothesized that lower neighbourhood income, remoteness, and variables reflecting potential service barriers such as proportions of immigrants and residents without knowledge of English or French would be associated with increased acute mental health service use. We also hypothesized that neighbourhood COVID-19 burden would also be associated with increased acute mental health service use because of pandemic-related stress and strain on local healthcare capacity.

## Methods

### Setting

We conducted a population-based study of all Ontario residents aged 0 to 24 between March 1, 2017, and July 31, 2021. Ontario is Canada's most populous province, representing approximately 40% of the national population and encompassing densely populated urban centres such as the Greater Toronto Area (approximately 6 million residents), mid-sized cities, and remote and rural regions, particularly in Northern Ontario (43). All residents receive publicly funded physician and hospital care.

Mental health services for children and youth are delivered through a combination of hospital-based and community-based programs that span childhood, adolescence and young adulthood. Hospital-based mental health care is available to individuals between the ages of 0 and 24 years, while community programs and Youth Wellness Hubs typically provide care to those aged 0 to 18 and 12 to 25 years, respectively. Therefore, to capture the full spectrum of child and youth mental health service use and to align with definitions of youth endorsed by the World Health Organization and United Nations, we studied individuals aged 0 to 24 years (44, 45).

We used aggregate dissemination areas (ADAs) as our primary geographic unit of analysis. ADAs are Canadian census units created by combining smaller census units, generally with a population of between 5,000 and 10,000 individuals. For descriptive purposes we use this term and neighbourhood synonymously.

### Data sources

We used Ontario's administrative health databases, linked using unique encoded identifiers and analyzed at ICES (formerly the Institute for Clinical Evaluative Sciences) in Toronto, Ontario (https://www.ices.on.ca). ICES is an independent, non-profit research organization and a prescribed entity under section 45 of Ontario's Personal Health Information Protection Act, which authorizes it to collect and analyze health information without individual consent or require review by a Research Ethics Board.

We identified mental health-related hospitalizations and ED visits using the Canadian Institute for Health Information Discharge Abstract Database (DAD), Ontario Mental Health Reporting System (OMHRS), and National Ambulatory Care Reporting System (NACRS) databases, The DAD contains clinical, demographic and administrative information from all admissions to acute care hospitals in Ontario. The NACRS contains similar information for visits to hospital- and community-based ambulatory care settings, including emergency departments, day surgery units, hemodialysis units, and cancer care clinics. The OMHRS contains information on all admissions to designated inpatient mental health beds in hospitals, provincial psychiatric facilities and specialty psychiatric facilities. We derived neighbourhood characteristics from the 2016 Census and determined the percent positivity of COVID-19 testing for each using the COVID-19 Integrated Testing (C19INTGR) database, which contains all SARS-CoV-2 PCR tests and results completed in Ontario.

### Outcomes

Our primary outcomes were rates of mental health-related hospitalizations and ED visits among children and youth (see Supplementary Table for diagnostic codes). For each neighbourhood, we calculated the rates of mental health-related hospitalizations and emergency department visits per 100,000 person-years children and youth.

### Neighbourhood characteristics

We used the 2016 Census to obtain neighbourhood sociodemographic characteristics that could be associated with the mental health of children and youth and access to health care. Specifically, we obtained the median neighbourhood income because pre-pandemic research has found associations with neighbourhood economic conditions and the mental health of children and youth. We also obtained three variables reflecting immigration and language, including the percentage of the population who were recent immigrants, visible minorities and without knowledge of English or French. We selected these variables as indicators of potential cultural and linguistic barriers to accessing care and cultural social support patterns that may influence help-seeking behaviour and mental health outcomes. To reflect family structure, we obtained measures of the average family size and the percentage of households that were multi-family dwellings. Average family size represents the number of persons per household and may reflect crowding and resource strain, whereas multi-family households may represent opportunities for shared caregiving and extended family support that could be protective during periods of stress. Finally, we obtained the proportion of the population employed in essential services as an indicator of the capacity to work from home during the pandemic, potentially influencing early recognition of mental health symptoms in children. We determined the neighbourhood COVID-19 burden using the C19INTGR database. We included this variable because we postulated that higher COVID-19 burden could influence the mental health of children and youth and associated help-seeking through increased family stress and potential strain on local healthcare capacity. To estimate neighbourhood walkability, we used the 2016 Canadian Active Living Environments (CAN-ALE) index, obtained from the Canadian Urban Environmental Health Research Consortium (46, 47). The CAN-ALE index is a composite measure summarizing the quality of the neighbourhood active living environment. It includes features such as intersection density (e.g., number of three-way or more intersections, offroad footpaths/recreational trails), dwelling density, and points of interest (e.g., parks, schools, shops, places of business, landmarks) within a one kilometer circular buffer around the center of each dissemination area (47). The CAN-ALE index is the sum of the z-scores of each measure, with negative, positive and zero values representing below average, above average, and average levels of walkability, respectively (47). We included this variable because of pre-pandemic research associating parks and green space with improved mental health outcomes in children and youth. Finally, because of mental health service gaps in remote and rural communities (48), we determined neighbourhood values for the Index of Remoteness, a continuous rurality measure ranging from 0 to 100, with zero and 100 representing the most urban and most remote areas, respectively (49). The Index of Remoteness is based on neighbourhood postal codes and represents the travel distance from a community to the closest population centres, defined as communities with greater than 1,000 individuals (49).

### Statistical analysis

Our analysis comprises two time periods. For the hot spot analysis (Research Question #1), we used data from March 2017 to July 2021 to enable comparison of the pre-pandemic (March 2017 to February 2020) and pandemic (March 2020 to July 2021) patterns of geographic distribution. For regression analyses examining neighbourhood correlates during the pandemic, (Research Questions #2 and #3), we used data from March 2020 to July 2021.

### Descriptive spatial analyses (research question #1)

We conducted hot spot analyses to identify clusters of mental health related hospitalizations and ED visits across Ontario neighbourhoods. The hot spot analysis was based on the GetisOrd (Gi*) statistic, a z-score that identifies clusters of points with values higher or lower in magnitude than would be expected by chance if the spatial distribution were random (50). We defined hot spots and cold spots as areas with relatively high and low acute mental health service use rates, respectively. To visualize changes in spatial patterns of acute mental health service use before and after the onset of the pandemic, we compared the distribution of these hot spots between the pre-pandemic (March 2017 to February 2020) and pandemic (March 2020 to July 2021) periods.

Next, we used multiple imputation by chained equations to impute missing values for seven neighbourhood characteristics, which ranged from 1.0% to 7.8% incomplete (51, 52). We examined associations between neighbourhood characteristics and service use with Pearson correlation coefficients and scatterplots, computing 95% confidence intervals using Fisher's z-transformation.

### Neighbourhood correlates of acute mental health service use during the height of the pandemic (March 2020 to July 2021) (research questions #2 and #3)

To assess the presence of spatial autocorrelation in the outcomes and covariates, we calculated the global Moran's *I* statistic (53, 54). The null hypothesis of Moran's *I* is that the observed spatial distribution is the result of random chance. Rejecting the null hypothesis indicates that the spatial distribution is either more clustered or dispersed than would be expected under a random process. We also calculated Moran's *I* for the residuals from an initial ordinary least squares regression (OLS) model to test for spatial autocorrelation in the residuals. Because significant spatial autocorrelation was detected in all variables and the OLS residuals, we used spatial autoregressive models with spatial lags of the dependent variable, independent variables and the error term (also known as spatial autoregressive with autoregressive error models, or SARAR models) to examine the associations between neighbourhood characteristics and mental health-related hospitalizations and emergency department visits among children and youth (53). We chose this specification because of the need to model dependence in outcomes, covariates and residual errors. We accounted for spatial dependence using an inverse distance weights matrix, whereby weights were assigned based on the inverse of the distance between neighbourhoods and then row-standardized so that the weights for neighbouring areas in each row summed to 1. We did not estimate alternative weight specifications.

Because we were primarily concerned with examining the relationships between neighbourhood characteristics and local mental health service use within the same neighbourhood, we applied row normalization to the spatial matrix. In this approach, the weights assigned to neighbouring areas are based on proximity, with the row-normalized weights in the matrix summing to 1, enabling the model to better capture localized effects (53, 54). Coefficients were then estimated to quantify both the direct effects of each independent variable on the outcome within the same neighbourhood and the indirect effects that reflect the ‘spillover’ impact of an independent variable in one neighbourhood on outcomes in adjacent neighbourhoods. We estimated models using non-standardized and standardized variables, with the latter allowing for a direct comparison of the relative strength of association between each neighbourhood characteristic and acute mental health service in standard deviation units. Standardized coefficients are particularly useful for comparing the effect sizes of variables measured on different scales. For instance, a standardized coefficient of 0.25 for median income in the hospitalization rate model suggests that a one standard deviation increase in neighbourhood median income is associated with a 0.25 standard deviation increase in the hospitalization rate.

### Sensitivity analyses

In a sensitivity analysis, we replicated our models using generalized spatial two-stage least squares (G2SLS) to assess the robustness of our findings to potential endogeneity and residual spatial autocorrelation (53, 55). G2SLS is a generalized method of moments approach that does not rely on strong distributional assumptions and is well-suited for data with unknown or non-normal distributions.

All analyses were completed using Stata version 18.0 (StataCorp LLC, College Station, TX, USA) and ESRI ArcGIS Map version 10.8 (ESRI, Redlands, CA, USA).

## Results

### Descriptive spatial analyses

**Figure 1:**
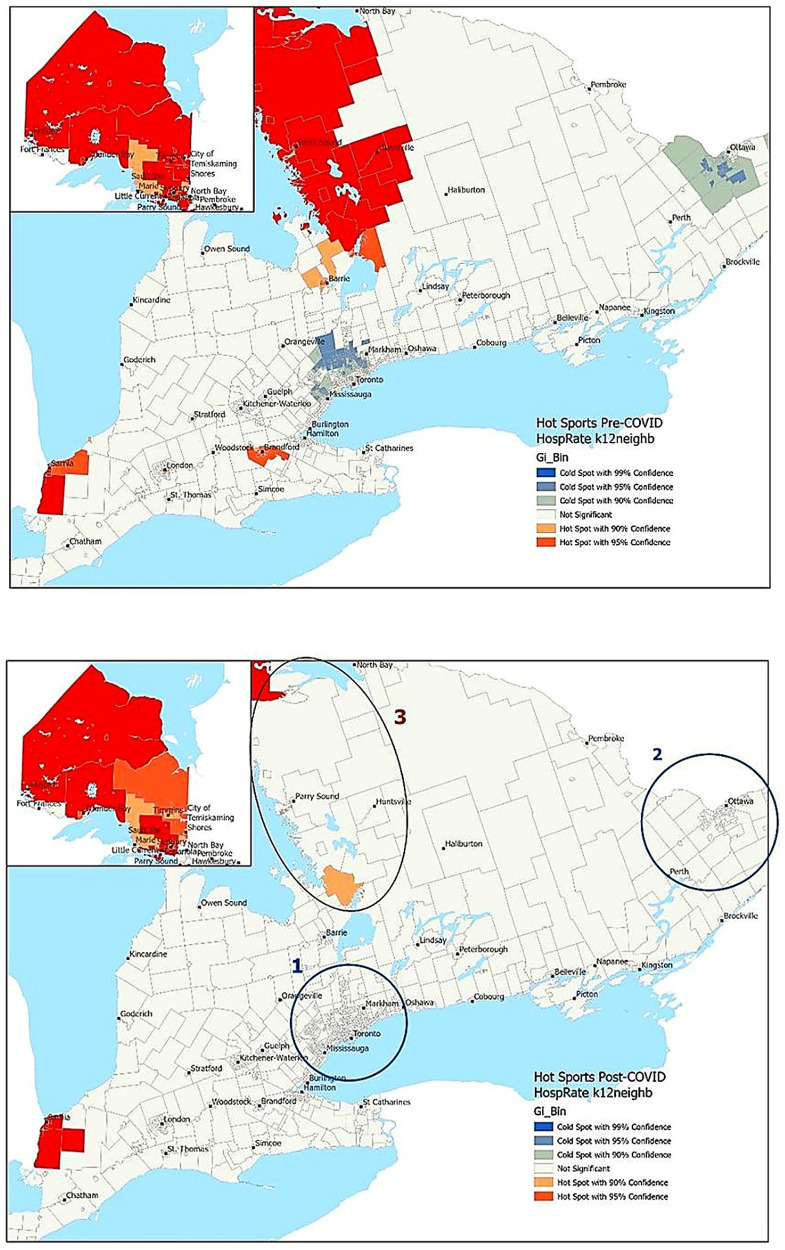
Pre- and post-pandemic hotspot analyses of mental health-related hospitalizations in children and youth. Large urban areas in the Greater Toronto Area and Ottawa were cold spots for mental-health-related hospitalizations in the pre-pandemic period. These cold spots disappeared following the pandemic, indicating an increase in hospitalization rates in these areas, though not enough to create new hot spots (areas 1 and 2). Northern regions of the province were persistent hot spots of mental health-related hospitalizations during the pre- and post-pandemic periods. However, the extent of these hot spots was reduced in some areas following the pandemic (area 3).

**Figure 2:**
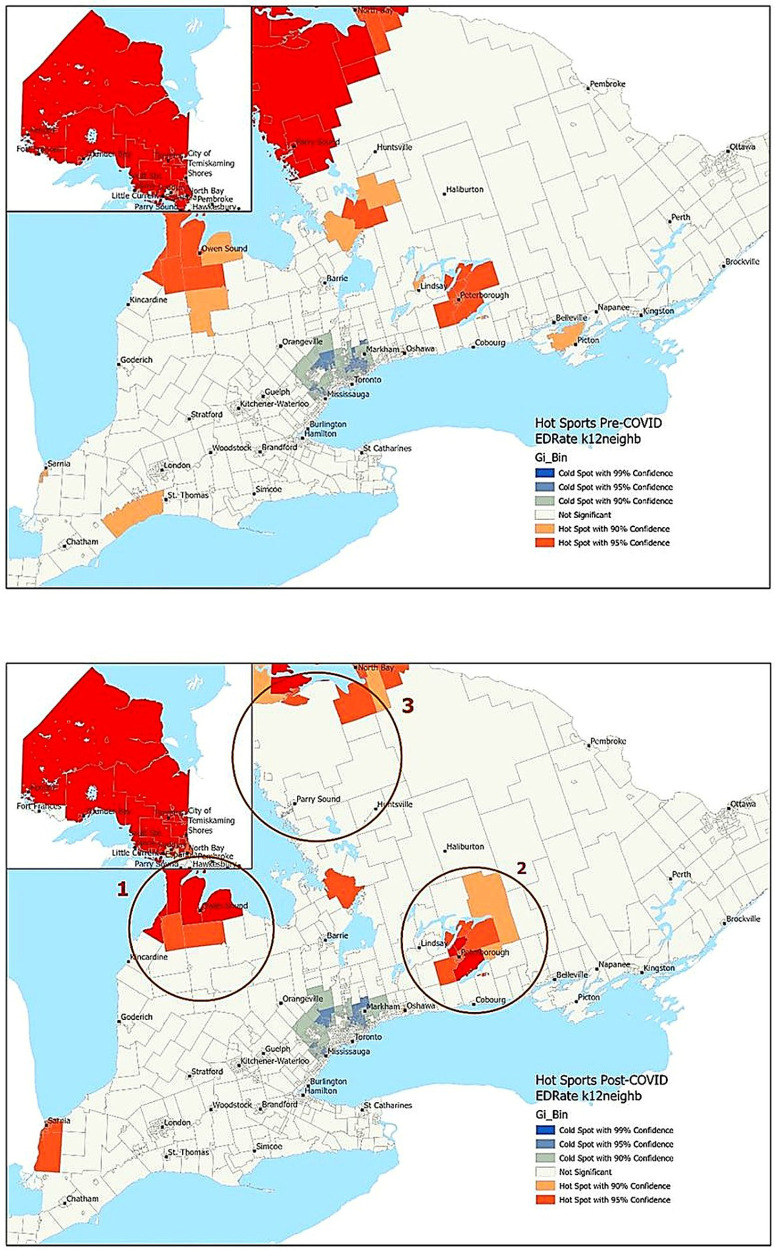
Pre- and post-pandemic hotspot analyses of mental health-related emergency department visits in children and youth. There was an increase in the number of hot spots in neighbourhoods around Owen Sound (Figure 2, Region 1) and Peterborough/Lindsay (Region 2), reflecting higher rates of mental health-related ED visits during the pandemic in these regions. In contrast, there was a decrease in hot spots in central-northern Ontario during the pandemic (Region 3), suggesting a redistribution of care or a relative decrease in ED use compared to neighbouring regions.

Hospitalization rates were highest in rural and northern Ontario, with lower rates observed in urban centres of the Greater Toronto Area (GTA) and Ottawa, as well as in some southern regions of the province (Figures 1, 2). The Greater Toronto Area (GTA) encompasses the city of Toronto and four surrounding regional municipalities (i.e., Durham, Halton, Peel, and York). This metropolitan area is one of Canada's most populous regions and includes both urban and suburban communities. The hot spot analysis identified northern regions of the province as persistent hot spots of mental health-related-hospitalizations during pre-pandemic and pandemic periods (Figure 1). However, the extent of these hot spots was reduced in some areas following the pandemic (Figure 1, area 3). While large urban areas in the GTA and Ottawa were cold spots for mental-health-related-hospitalizations in the pre-pandemic period, these cold spots disappeared during the pandemic, indicating an increase in hospitalization rates in these areas, though not enough to create new hot spots (Figure 1, areas 1 and 2).

Similar patterns were observed for ED visits, with lower rates seen in urban centers and higher rates in northern and central Ontario (Figure 2). In hot spot analyses, persistent cold spots were identified in the GTA before and after the pandemic (Figure 2). However, there was an increase in the number of hot spots in outlying neighbourhoods around Owen Sound (Figure 2, Region 1) and Peterborough/Lindsay (Figure 2, Region 2), reflecting higher rates of mental health-related ED visits during the pandemic. In contrast, there was a decrease in hot spots in central-northern Ontario (Figure 2 Region 3) during the pandemic, suggesting a redistribution of care or a relative decrease in ED use compared to neighbouring regions.

In correlation analyses, neighbourhood income was inversely correlated with most neighbourhood characteristics, reflecting a higher prevalence of essential workers (*r* = −0.33), COVID positivity (*r* = −0.29) and individuals without knowledge of English or French (*r* = −0.36) in the lowest income neighbourhoods (Supplementary Figure S1). Remoteness was negatively correlated with the CAN-ALE (*r* = −0.29), suggesting that walkable neighbourhoods are generally located in urban settings. Strong negative correlations were observed between income and mental health-related hospitalization and ED rates, while remoteness correlated positively with both outcomes (Supplementary Figures S2, S3).

### Correlates of mental health-related hospitalization

**Figure 3:**
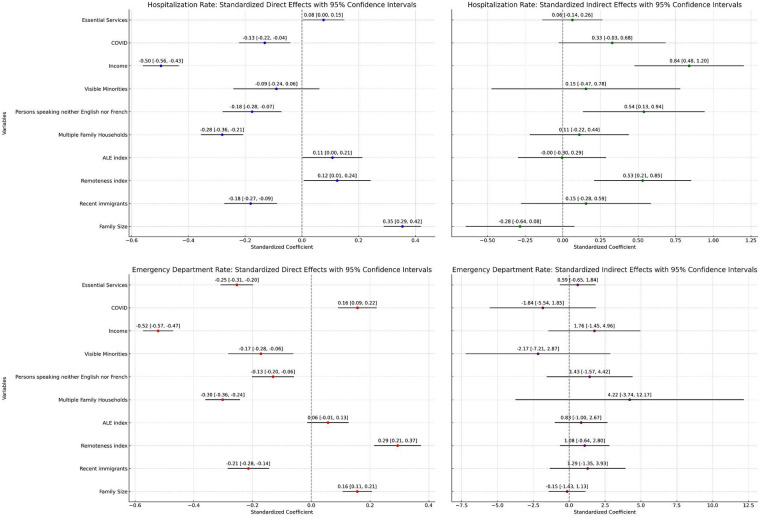
Standardized coefficients for mental health-related hospitalizations and emergency department visits in Ontario children and youth.

The mean and median rates of mental health-related hospitalizations across neighbourhoods, weighted by population size, were 494.9 [standard deviation (SD) ± 353.6] and 408.8 [interquartile range (IQR): 324.0] per 100,000 person-years. Following multivariable spatial regression analysis, several variables were associated with significant local (i.e., direct) effects on mental health-related hospitalizations within the same neighbourhood (Table 1). Specifically, mental health-related hospitalizations declined by 73.6 per 100,000 person-years for each $1000 increase in median neighbourhood income. In addition, each percentage point increase in the proportion of the population whose first language was neither English nor French, the proportion of the population who were recent immigrants, and the proportion of households that were multi-family dwellings were associated with declines of 56.1 (95% CI: −89.6 to −22.6, *p* = 0.001), 53.9 (95% CI: −81.1 to −26.8) and 73.5 (95% CI: −92.6 to −54.4) hospitalizations per 100,000 person-years, respectively. Moreover, each percentage point increase in COVID-19 test positivity was associated with a slight decrease in the local hospitalization rate (−0.07 per 100,000 person-years; 95% CI: −0.11 to −0.02), indicating a modest absolute effect. Conversely, increases in average family size, index of remoteness, the percentage of the population employed in essential services, and the CAN-ALE index were associated with increases in hospitalization rates, with the largest absolute increases observed for family size and remoteness (Table 1). Following variable standardization, median neighbourhood income and average family size were the variables most strongly associated with mental health-related hospitalizations within the same neighbourhoods (Figure 3). Specifically, a one standard deviation increase in median income was associated with a 0.50 standard deviation decrease in hospitalization rates (95% CI: −0.56 to −0.43), while a one standard deviation increase in average family size was associated with a 0.35 standard deviation increase in hospitalization rates (95% CI: 0.29 to 0.42).

**Table 1:** Neighbourhood correlates of mental health-related hospitalizations and emergency department visits in Ontario children and youth, March 2020 to July 2021.

Neighbourhood characteristic	Hospitalizations		Emergency department visits	
	Direct effects (95% confidence intervals)	Indirect effects (95% confidence intervals)	Direct effects (95% confidence intervals)	Indirect effects (95% confidence intervals
Median Income (per $1000 Increase)	−73.6 (−82.9 to −64.2)	147.0 (87.5 to 206.6)	−172.1 (−189.1 to −155.1)	579.5 (−476.1 to 1635.0)
Persons without knowledge of English or French	−56.1 (−89.6 to −22.6)	204.6 (61.4 to 347.9)	−95.7 (−148.2 to −43.2)	1,048.1 (−1,150.4 to 3246.6)
Recent Immigrants	−53.9 (−81.1 to −26.8)	48.8 (−86.9 to 184.6)	−140.6 (−186.9 to −94.3)	844.8 (−886.5 to 2576.2)
Visible Minorities	−3.4 (−9.2 to 2.4)	9.9 (−15.0 to 34.8)	−14.8 (−24.4 to −5.3)	−187.9 (−623.5 to 247.7)
Persons Employed in Essential Services	7.9 (0.32 to 15.5)	1.48 (−23.98 to 26.78)	−59.2 (−72.2 to −46.3)	138.9 (−151.8 to 429.5)
Average Family Size	1,386.8 (1,132.3 to 1641.3)	−1513.4 (−3,037.8 to 10.9)	1,366.0 (930.2 to 1,801.8)	−1,300.1 (−12483.9 to 9,883.7)
Multiple Family Households	−73.5 (−92.6 to −54.4)	−2.0 (−99.1 to 95.1)	−174.4 (−208.5 to −140.2)	2,434.2 (−21,609 to 7,029.1)
Percent COVID Test Positivity	−0.07 (−0.11 to −0.02)	0.24 (0.05 to 0.44)	0.18 (0.10 to 0.25)	−2.1 (−6.3 to 2.2)
Active Living Environments Index	31.5 (0.48 to 62.5)	1.2 (−84.9 to 87.0)	38.6 (−9.5 to 86.7)	566.0 (−682.2 to 1814.1)
Index of Remoteness	937.5 (118.8 to 1,756.2)	4,989.3 (2,177.1 to 7,801.4)	4,656.5 (3,392.7 to 5,920.3)	17,098.4 (−10,148.6 to 44,345.4)

In addition to local effects, several variables exhibited significant spillover (i.e., indirect) effects on mental health-related hospitalizations in adjacent neighbourhoods (Table 1). Specifically, one-unit increases in remoteness and the percentage of the population without English or French in one neighbourhood were associated with increased hospitalization rates in adjacent areas (Table 1). Similarly, a $1,000 increase in median income was associated with an increase of 147.0 hospitalizations per 100,000 person-years (95% CI: 87.5 to 206.6) in neighbouring areas. Following variable standardization, median neighbourhood income, the proportion of the population speaking neither English nor French, and remoteness were associated with the strongest spillover effects (Figure 3). A one standard deviation increase in each of these variables was associated with respective increases in the standard deviation of hospitalization rates of 0.84 (95% CI: 0.48 to 1.20), 0.54 (95% CI: 0.13 to 0.94), and 0.53 (95% CI: 0.21 to 0.85) in neighbouring areas.

Results were generally similar when using the G2SLS estimator in terms of both the direction and magnitude of the direct and indirect effects, though the spillover effect for average family size (−1675.9 hospitalizations per 100,000; 95% CI: −2830.0 to −521.9) was significant with the change in estimation approach (Supplementary Table S2).

### Correlates of mental health-related Ed visits

The mean and median rates of emergency department visits across neighbourhoods, weighted by population size, were 1584.5 (SD ± 1149.8) and 1287.2 (IQR: 1026.9) per 100,000 person-years, respectively. Several variables were associated with significant local effects on mental health-related ED visits within the same neighbourhood (Table 1). Each $1000 increase in median neighbourhood income was associated with a decrease in ED visit rates of 172.1 visits per 100,000 person-years (95% CI: −189.1 to −155.1). Higher percentages of persons employed in essential services, visible minorities, recent immigrants, persons without knowledge of English or French and multiple-family households were also associated with reductions in ED visit rates (Table 1). Conversely, greater family size and remoteness were associated with increases in ED visit rates of 1366.0 (95% CI: 930.2 to 1801.8) and 4656.5 (95% CI: 3392.7 to 5920.3) per 100,000 person-years for each unit increase in the value of these variables. In an analysis of standardized variables, median neighbourhood income was most strongly associated with mental health-related ED visits, with each standard deviation increase in income associated with a 0.52 standard deviation decrease in ED visits (95% CI: −0.57 to −0.47) (Figure 3). The strongest positive association was with remoteness, with local ED visit rates increasing by 0.29 (95% CI: 0.21 to 0.37) standard deviations for each standard deviation increase in the remoteness index (Figure 3).

No significant spillover effects were observed, suggesting that changes in neighbourhood characteristics in one region did not influence ED visit rates in neighbouring regions. In sensitivity analyses, findings from the G2SLS model were generally aligned with those obtained using the maximum likelihood estimator (Supplementary Table S2).

## Discussion

In our population-based study, we found substantial variation in rates of mental health-related emergency department visits and hospitalizations among Ontario children and youth during the COVID-19 pandemic. This variation was influenced by several neighbourhood characteristics reflecting local socioeconomic and demographic conditions, with the most pronounced effects imparted by neighbourhood income, family size, remoteness and the prevalence of multi-family households. Furthermore, several variables exhibited spillover effects for hospitalizations, indicating complex spatial dependencies between neighbouring regions. Our findings were robust to changes in the estimator, as results from G2SLS were broadly consistent with those obtained from maximum likelihood estimation. The main difference with the G2SLS sensitivity analysis was that the spillover association between average family size and hospitalization rates became statistically significant, although the magnitude and direction of effect were similar. This difference reflects the estimators’ distinct approaches to modelling spatial autocorrelation.

Median household income was the neighbourhood characteristic most strongly associated with acute mental health service use by children and youth. In addition, we also observed spillover effects of neighbourhood income, in which higher income in one neighbourhood was associated with increased hospitalization rates in adjacent neighbourhoods. Several mechanisms may explain this finding that relate to within neighbourhood advantages and gradients between neighbourhoods. First, the inverse association between neighbourhood income and acute mental health services within the same neighbourhood suggests that children and youth in higher-income neighbourhoods were better positioned to manage pandemic-related mental health challenges than those in lower-income neighbourhoods. This may reflect disparities in the availability of mental health resources and specialized care favouring higher income areas. Past research has found socioeconomic gradients in the receipt of publicly-funded psychiatric care (56–58). Moreover, higher income families have the financial resources to access services not covered by the publicly-funded health care system in Ontario, such as psychologists and social workers. Approximately 30% of Canadians are estimated to pay out of pocket for such services, representing a significant barrier to accessing mental health services for low-income families (59).

Second, higher-income neighbourhoods also generally exhibit lower crime rates and better housing conditions than lower-income neighbourhoods, both of which are protective factors for the mental health of children and youth (60, 61). Exposure to the negative aspects of the neighbourhood-built environment may have increased for children and youth in low-income neighbourhoods because of stay-at-home orders and school closures, potentially contributing to mental health symptoms and acute mental health service needs (62).

Third, the spillover phenomenon may reflect between neighbourhood gradients in the rapid transition to virtual mental health care at the outset of the pandemic and socioeconomic disparities in the ability to work from home, both of which favoured families in high-income neighbourhoods and which was associated with a paradoxical spillover effect (63–66). Specifically, an income gradient was observed in the delivery of virtual mental health services, with higher-income families having better access to the technology, flexibility and resources needed to schedule and attend virtual mental health appointments (64, 66). Parental presence at home may have also facilitated earlier recognition of mental health symptoms in children and youth, increasing opportunities for intervention before reaching crisis levels. In contrast, lower-income parents, often employed in essential in-person jobs, had less flexibility and fewer resources to recognize and address mental health issues early, potentially delaying care and contributing to higher hospitalization rates. Overall, these findings underscore the local and broader influence of neighbourhood income on acute mental health service use during the pandemic, highlighting the need to reduce income-related barriers to community and virtual mental health care in lower-income neighbourhoods.

These same dynamics could explain lower ED visit rates but higher hospitalization rates as the percentage of essential workers within a neighbourhood increased. Parents employed in essential service jobs would have fewer opportunities to observe changes in the mental health of their children, potentially delaying recognition of symptoms until hospitalization became necessary (67, 68). Furthermore, the adverse effects of pandemic-associated school closures and disruptions in routine were amplified for children of essential workers by the additional stress of parental exposure to COVID-19 and high rates of mental health symptoms and suicidal ideation reported among essential worker parents (69–71).

We also observed associations between average family size and the prevalence of multiple family households and acute mental health service use, with the former associated with increased local hospitalization and ED visit rates and the latter exerting protective effects. Although both concepts relate to family dynamics within neighbourhoods, they reflect different phenomena with distinct implications for the mental health of children and youth. Larger family size typically refers to larger nuclear families, generally representing households with more children but fewer adults to share caregiving responsibilities (72). Past research has linked household chaos with the number of people in the home and parental stress, contributing to adverse mental health and behavioural outcomes in children (73–79). Furthermore, chaotic home environments appear to mediate the relationship between socioeconomic status and mental health outcomes in children and youth through several mechanisms, including parental mental health and family stress (77–79). These effects may have been compounded during the pandemic, contributing to a higher risk of mental health crises among children and youth (80). Conversely, multiple-family housing refers to households where multiple families or extended family members cohabit (81). Such arrangements typically involve multiple adult caregivers and sharing of resources, thereby potentially providing social and economic buffers against the harmful effects of the pandemic that were not available to larger nuclear families lacking the support of an extended family network (82, 83). This aligns with findings that multiple family living arrangements increased during the pandemic, particularly among low-income families seeking to pool resources and cope with economic and childcare challenges (84, 85). Alternatively, lower rates of acute mental health service use in neighbourhoods with more multiple-family housing could reflect language and cultural barriers to accessing care, or higher thresholds for seeking hospital-based services rather than a lower need for care. Because of the nature of our study, we are unable to definitively identify the causal mechanism underlying the association between multiple family housing and lower hospitalization and ED visit rates. Further research is required to distinguish protective effects from access barriers. Taken together, the associations with family size and multiple-family households suggest that the effects of household structure are likely heterogenous and mediated by protective factors, stress, and barriers to care.

Neighbourhood remoteness was associated with increased acute mental health service use within the same neighbourhood, with increased spillover effects observed for hospitalizations. This finding aligns with past research highlighting long-standing shortages of mental health expertise in rural and remote regions, resulting in longer wait times and a need to travel great distances to access specialized care (48). In 2020, 28,000 Ontario children and youth were waiting up to 2.5 years for mental health services, with those in rural and remote communities being especially disadvantaged and having to travel to large urban centres for intensive treatment (86). Similar disparities exist in the mental health workforce. In Canada, the number of psychiatrists per 100,000 population is 17 and 3 in urban and rural regions, respectively, with similar clustering observed for other mental health professionals (48). An Ontario study found that 40% of psychiatrists worked in a densely-populated urban centre that is home to 10% of the province's population (87). The effects of these pre-existing workforce disparities were likely exacerbated by pandemic-associated disruptions in local outpatient and community-based mental health services (88), with emergency departments and hospitals becoming the primary response mechanism for children and youth with mental health symptoms in these areas. The spillover effects to hospitals in neighbouring regions could represent a transfer of children and youth to larger urban centres where the majority of psychiatric hospitals and mental health beds are clustered. Additionally, the positive spillover effect to adjacent neighbourhoods may have occurred because of capacity constraints among local hospitals overwhelmed by COVID-19 cases, limiting capacity for psychiatric care. As a result, hospitals in nearby areas with fewer COVID patients or better capacity to handle psychiatric admissions may have absorbed the demand for mental health services. These findings reinforce longstanding calls to expand child and youth mental health care in rural and remote regions.

A similar mechanism could explain the effects of neighbourhood COVID positivity. In Ontario, as in other jurisdictions, emergency protocols were implemented enabling the transfer of COVID-19 and non-COVID-19 patients from overwhelmed hospitals to those with available capacity, including children and youth (89, 90). In addition, several measures were undertaken across the province to protect the capacity for caring for COVID-19 patients, including redeployment of staff, cancellation of non-urgent clinical activity and repurposing pediatric inpatient beds to create additional adult inpatient capacity (91, 92). This process could explain the decrease in local hospitalizations for mental health conditions as COVID positivity increased and the corresponding increase in hospitalizations in adjacent neighbourhoods, reflecting the transfer of patients.

Local rates of mental health-related acute mental health service use declined in neighbourhoods with increased populations of individuals who spoke neither English nor French and recent immigrants. This finding is consistent with past research demonstrating an association between language barriers and accessing psychiatric care and suggests that these populations may have faced language and cultural barriers to accessing acute mental health care for their children within their neighbourhoods (93). The positive spillover effects observed for hospitalization among non-English/French speakers may reflect patterns of healthcare-seeking behaviors wherein individuals travel to hospitals in adjacent neighbourhoods known to offer more linguistically appropriate services, particularly given the findings of past research associating language discordant encounters with negative health outcomes (94). Community networks or word-of-mouth recommendations may guide individuals to hospitals with established interpreter services or multilingual providers better equipped to accommodate the needs families who speak neither official language (95). Further research is needed to understand the basis of this finding. Among families who are recent immigrants to Canada, language barriers to accessing care are compounded further by a lack of familiarity with a new healthcare system. However, the inverse association between acute mental health service use and recent immigration may reflect less need for such care among the children of families who are recent immigrants (96). This assertion is supported by past research demonstrating positive mental health outcomes among immigrant children and youth with increasing neighbourhood immigrant concentration, an association that may be mediated by shared cultural norms, supportive community processes and relationships, and collective efficacy marked by a willingness of residents to intervene on behalf of children (96, 97). Furthermore, past research has found that the adverse effects of family poverty on externalizing behavior problems are attenuated among children of recent immigrants (96). Thus, in addition to the possibility of reduced access to linguistically- and culturally-appropriate services, the inverse association between acute mental health service use and the recent immigrant population may represent heightened resilience to stress among these families. Finally, an Ontario study found that children of immigrant families had higher than expected rates of virtual pediatric mental health visit rates during the first year of the pandemic, suggesting that these children may have been able to access to the care required on an outpatient basis (66). Overall, these findings suggest that a complex interplay exists between barriers to linguistically and culturally appropriate care and potential resilience among recent immigrants and non-official language speakers. Access to culturally responsive mental health services and multilingual capacity is needed to facilitate help seeking among immigrant and ethnoculturally diverse communities.

The association between neighbourhood walkability and increased hospitalizations within the same neighbourhood is perhaps counterintuitive, given past research showing that walkable neighbourhoods are associated with improved mental health outcomes. However, as demonstrated by the negative correlation between the ALE index and remoteness, walkable neighbourhoods are generally located in urban or suburban settings with more plentiful services, amenities, and healthcare infrastructure. Approximately 20% of Canadians are estimated to live in amenity-dense neighbourhoods characterized by proximity to a variety of services, including healthcare (98). Moreover, most Canadians living in larger metropolitan areas live within a 3 kilometer drive to a healthcare facility, compared with only half of rural residents (98). Furthermore, as noted earlier, mental health expertise and facilities with designated mental health inpatient beds are clustered in large urban regions (48). Thus, the increased proximity to healthcare may partly explain the higher hospitalization rates observed in walkable neighbourhoods. However, we did not have direct data on the distribution or capacity of mental health facilities, rendering this interpretation speculative and one of several possible explanations.

Our findings have important implications for practice and policy, particularly in the context of preparing for future large-scale disruptions. Most notably, our findings highlight the importance of long-term solutions that strengthen mental health services and address the intersectional nature of challenges faced by children and youth during periods of widespread uncertainty and crisis. Such measures could include incentives for attracting mental health professionals to remote regions and low-income neighbourhoods, increasing the number of acute mental health beds in underserved areas to minimize the need for emergency transfers to adjacent better resourced areas, and expanding virtual and telepsychiatry services. Expanding digital infrastructure in low-income and remote communities and providing technology subsidies to low-income families are important considerations for ensuring that investments in virtual mental health do not inadvertently exacerbate existing disparities. Optimizing the uptake of programs such as the United States Affordable Connectivity Program and the Canadian Connecting Families Initiative is needed to ensure equitable access to virtual care. Furthermore, although interpretation services are available in hospitals within large urban centers such as Toronto, standardizing access to these programs across the province and ensuring that staff are aware of the availability of these services is required to address barriers related to the access to linguistically congruent care (99, 100).

In addition to long-term measures, our findings also emphasize the need for rapid interventions that can be scaled up during future large-scale disruptions. Rates of acute mental health service use increased with family size, suggesting that emergency deployment of financial and social programs is needed to support large families in times of stress. Although programs such as the Canada Emergency Response Benefit (CERB) were rapidly deployed to support individuals who suddenly lost employment during the pandemic, the expansion of such benefits to low-income and large families, regardless of employment status, could be considered a future policy initiative to mitigate the financial stresses of future disruptions. A survey of CERB recipients supports such a future expansion, with most respondents describing the program as a source of stability for their household's financial situation and positively impacting their mental health (101). However, some respondents expressed that the funding was insufficient to meet the needs of more than one person, underscoring the importance of scaling future emergency benefits programs to account for dependents and family size (101).

Children of essential workers were also vulnerable to hospitalization for mental health conditions. Psychological first aid programs tailored to the needs of essential workers and their children and delivered within the first days of future pandemics or disruptions represent one approach to supporting these families (102). Providing publicly-funded counselling and psychology services, even temporarily, is another measure for expanding mental health care access to children of essential workers and low-income children. Our data also suggests that measures are needed to minimize transfers of children and youth to hospitals in adjacent neighbourhoods. Rapid scale-up of mobile mental health units and crisis intervention teams in areas lacking inpatient mental health care capacity and neighbourhoods heavily impacted by COVID are potential measures for mitigating the needs of such measures.

Our study has several limitations. First, our study covers only the first 1.5 years of the pandemic, limiting our ability to assess changes in neighbourhood correlates of acute mental health service use over time. However, this limitation must be balanced against the population-based nature of our study, our comprehensive identification of emergency department visits and hospitalizations, and the use of spatial regression methods that quantify local and spillover effects of neighbourhood characteristics on acute mental health service use. Our study therefore builds upon past research describing individual- and family-level correlates of mental health in children and youth and studies ascertaining mental health symptoms using parent or child self-report (24). Second, we did not have access to neighbourhood characteristics known to influence the mental health of children and youth, such as community crime rates and neighbourhood social capital (28–36). In addition, we did not have access to potential mediators of the relationship between neighbourhood and mental health, such as parenting behaviour, family conflict and family resources, thereby limiting our ability to fully explain the mechanisms underlying our findings. Further qualitative and quantitative research is needed to examine these mechanisms and inform programming and policy. However, our findings provide clinicians and policymakers with important insights into neighbourhood features that may predict hot spots of acute mental health service need in future pandemics, including how specific neighbourhood factors like income, remoteness, and COVID positivity influenced mental health service use beyond their immediate boundaries and the nature of interventions needed to support families during future disruptions. Third, our study included individuals aged 0 to 24 years, consistent with the age range served by Ontario's hospital-based mental healthcare system for acute psychiatric care and definitions of youth endorsed by the WHO and UN (44, 45). However, this broad age range did not allow us to examine whether associations between neighbourhood characteristics and acute mental health use differed across childhood, adolescence, and emerging adulthood. Similarly, we did not examine whether neighbourhood correlates of acute mental health service use differed between females and males. Past research has documented differences in mental health service use by sex, potentially modifying associations with neighbourhood characteristics (103). Future studies using age- and sex-stratified analyses could clarify whether neighbourhood determinants differ by developmental stage or sex. Finally, our study was conducted in a single Canadian province with publicly funded health care and may not be generalizable to other settings.

In conclusion, we identified several neighbourhood correlates of acute mental health service use in children and youth during the COVID-19 pandemic. Together, these findings highlight the importance of neighbourhood context in determining mental health outcomes and access to care during periods of widespread disruption. Building more equitable and resilient systems to support child and youth mental health during future crises will require coordinated action across three levels. First, clinical care services are needed that proactively target high-risk areas and address cultural and linguistic barriers to seeking help. Second, health system organization should ensure adequate geographic distribution of resources and capacity. Third, social policy is needed that addresses underlying neighbourhood determinants of health through investments in economic security, housing, childcare, and community infrastructure. In addition, these findings emphasize the importance of preparing for large-scale disruptions in vulnerable neighbourhoods with proactive strategies that strengthen mental health access and the rapid deployment of targeted interventions that prevent escalation of mental health crises among children and youth during future pandemics or widespread disruptions.

## Data Availability

The data analyzed in this study is subject to the following licenses/restrictions: The data set from this study are held securely in coded form at ICES. While data sharing agreements prohibit ICES from making the data set publicly available, access may be granted to those who meet pre-specified criteria for confidential access, available at www.ices.on.ca/DAS.
