# Supplementary material for: Geographic variation and neighbourhood correlates of mental health-related hospitalizations and emergency department visits in children and youth during the COVID-19 pandemic: a population-based study
Source: Front Child Adolesc Psychiatry. 2026 May 18;5:1575531. doi: 10.3389/frcha.2026.1575531 (PMC13222987; doi:10.3389/frcha.2026.1575531)
Supplement: Supplementary file 1 [file Supplementaryfile1.docx]

Supplemental Table 1: Diagnostic codes for ascertaining mental health-related hospitalizations and emergency department visits

| **Clinical category** | **ICD-9-CM codes (OMHRS DSM 5)** | ​​**ICD-10-CA codes (DAD/NACRS)** |
| --- | --- | --- |
| **Any mental health and addictions** | - DSM5CODE_DISCH1 = Any OMHRS (includes missing; excludes 290.x, 294.0x-). - Exclude if DSM5CODE_DISCH1 missing and Provisional =17 | DX10CODE1= F06-F99 or DX10CODE2-DX10CODE10 = X60-X84, Y10-Y19, Y28 when DX10CODE1 ne F06-F99 |
| **Substance-Related and Addictive Disorders** | - DSM5CODE_DISCH1 = 291.x (all 291 codes), 292.x (all 292 codes), 303.x (all 303 codes), 304.x (all 304 codes), 305.x. - ​​ Provisional =16 | DX10CODE1 = F10-19, F55 |
| **Schizophrenia Spectrum and Other Psychotic Disorders** | - DSM5CODE_DISCH1 = 293.81, 293.82, 295.x (all 295 codes), 297.x (all 297 codes), 298.x (all 298 codes). - Provisional =2 | DX10CODE1 = F06.0-2, F20, F22-F29, F53.1 |
| **Mood disorders** | - DSM5CODE_DISCH1 = 293.83, 296.x (all 296 codes), 300.4x, 301.13, 311.x, 625.4. Provisional =3, 4 | - DX10CODE1 = F06.3, F30.x-F34.x, F38.x, F39.x, F53.0 |
| **Anxiety disorders*** | - DSM5CODE_DISCH1 = 293.84, 300, 300.0x, 300.2x, 309.21, 313.23. - Provisional = 5 | DX10CODE1 = F06.4, F40, F41, F93.0-2, F94.0 |
| **Trauma/stressor-related disorders** | - DSM5CODE_DISCH1 = 308.3x, 309, 309.0x, 309.24, 309.28, 309.3x, 309.4x, 309.81, 309.89, 309.9x, 313.89. - Provisional = 7 | DX​10CODE1 = F43.x, F94.1, F94.2 |
| **OCD & related disorders** | - DSM5CODE_DISCH1 = 300.3x, 300.7x, 312.39, 698.4x. - Provisional = 6 | DX10CODE1 = F42.x, F45.2, F63.3 |
| **Personality disorders** | - DSM5CODE_DISCH1 = 301, 301.0x, 301.2x, 301.4x, 301.5x, 301.6x, 301.7x, 301.81-3, 301.89, 301.9x 310.1. - Provisional = 18 | DX10CODE1 = F07, F21, F60, F61, F62. F68, F69 |
| **Deliberate self-harm**† | N/A (DAD/NACRS only) | DX10CODE2-10 (NACRS)/DXCODE2-25(DAD) = X60-X84, Y10-Y19, Y28 when DX10CODE1 ne F06-F99 |

**Supplemental Figure 1: Correlation Between Neighbourhood Characteristics**


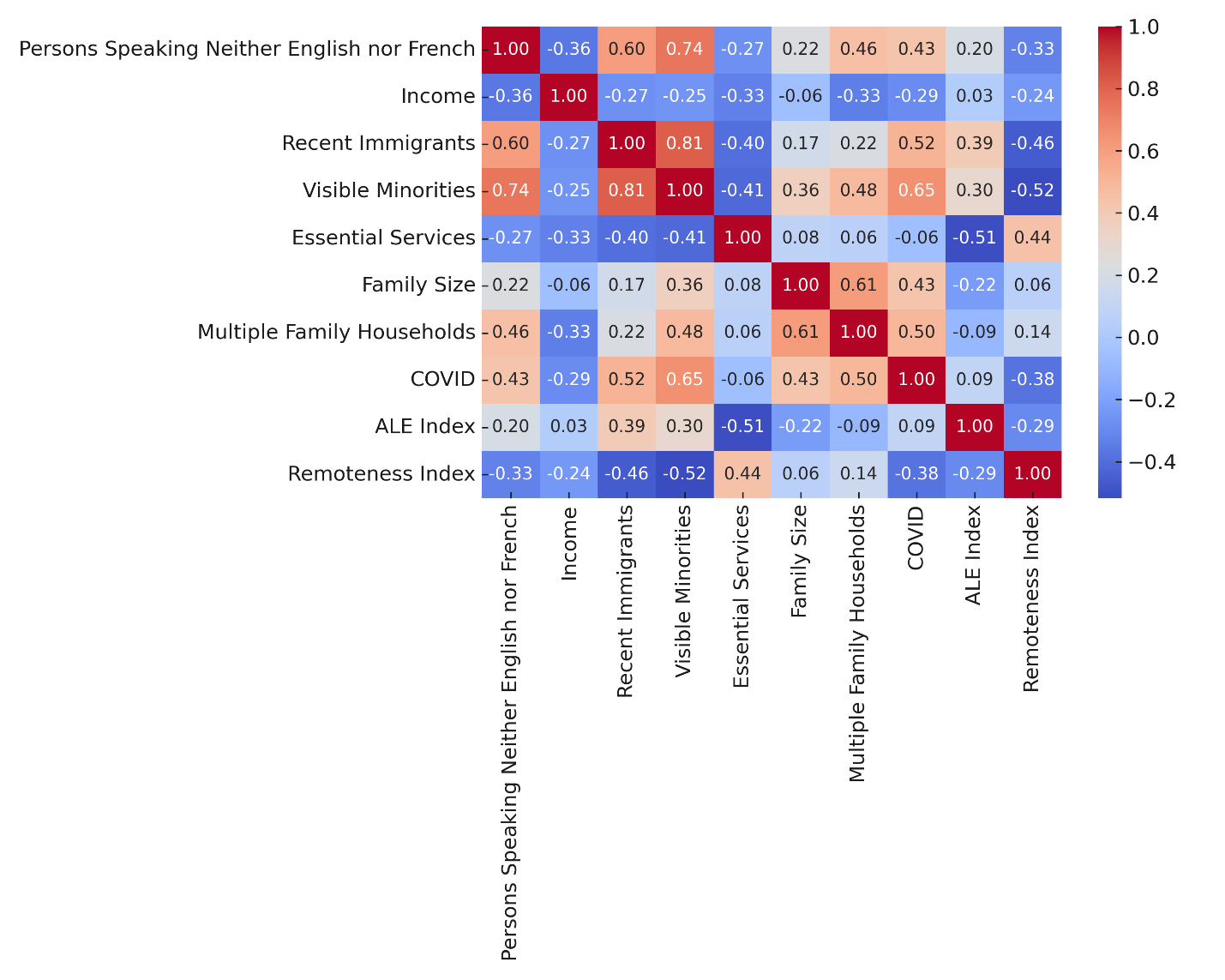


**Supplemental Figure 2: Correlation Between Neighbourhood Characteristics and Mental Health-Related Hospitalizations among Children and Youth**


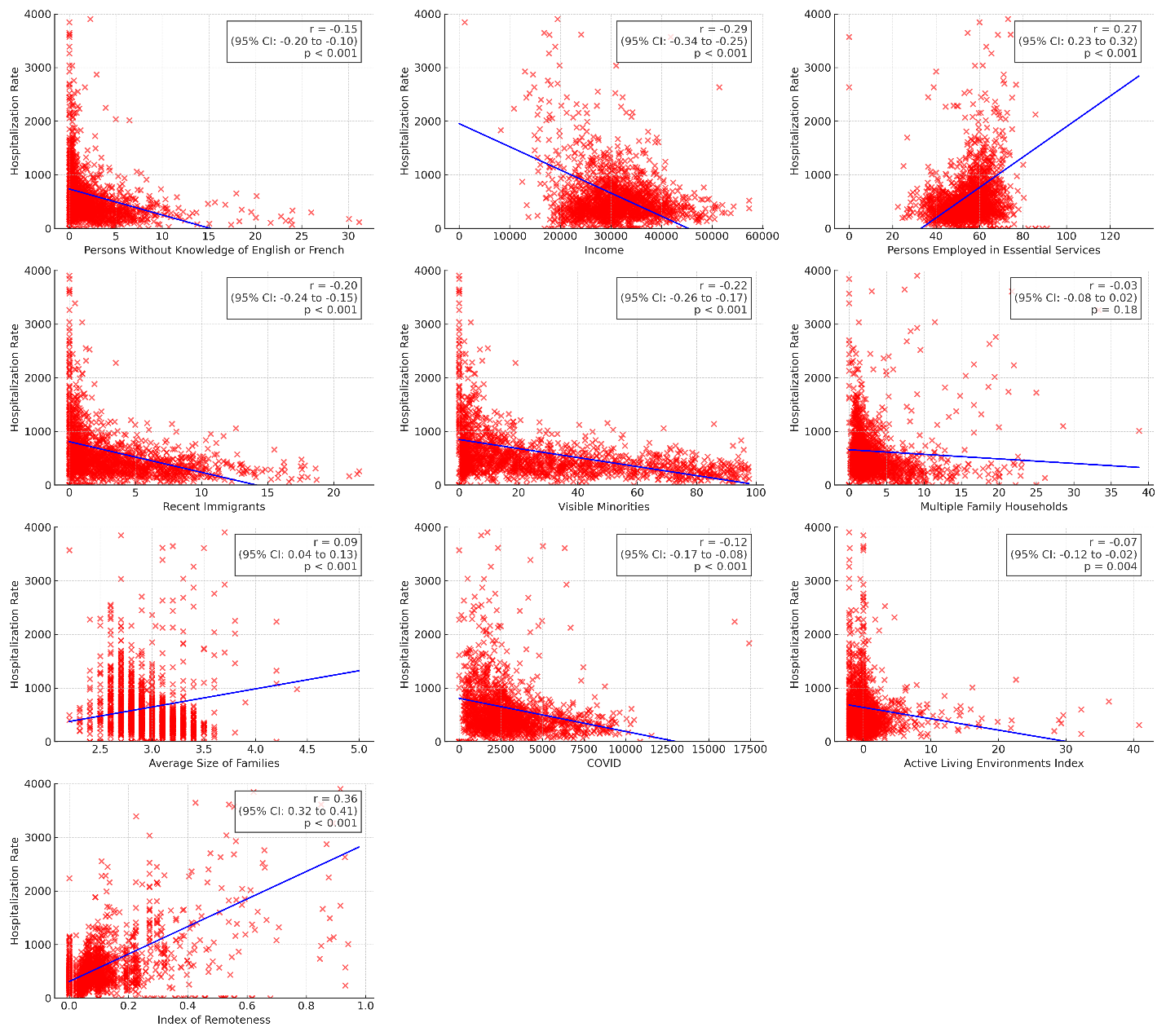


**Supplemental Figure 3: Correlation Between Neighbourhood Characteristics and Mental Health-Related Emergency Department Visits Among Children and Youth**


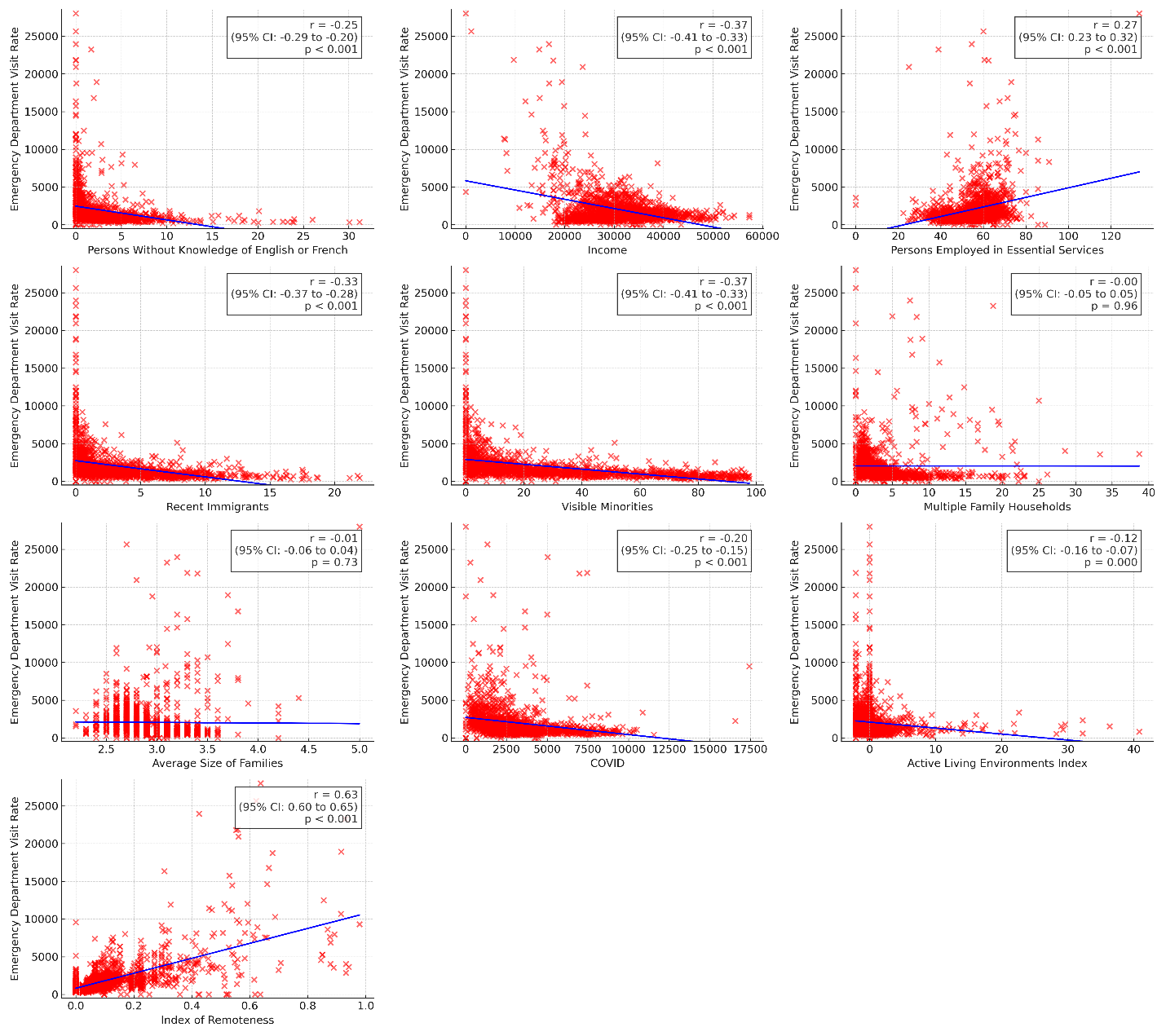


**Supplemental Table 2: Sensitivity Analysis of Neighbourhood Correlates of Mental Health-Related Hospitalizations and Emergency Department Visits in Ontario Children and Youth, March 2020 to July 2021**

|  | **Hospitalizations** | | **Emergency Department Visits** | |
| --- | --- | --- | --- | --- |
|  | Direct Effects (95% Confidence Intervals) | Indirect Effects (95% Confidence Intervals) | Direct Effects (95% Confidence Intervals) | Indirect Effects (95% Confidence Intervals |
| Median Income (per $1000 Increase) | -71.6 (-81.4 to -61.9) | 108.7 (62.4 to 155.0) | -169.9 (-186.8 to -153.0) | 1826.6 (-804.4 to 11697.6) |
| Persons without knowledge of English or French | -42.4 (-75.6 to -9.2) | 113.1 (7.1 to 219.0) | -89.9 (-140.3 to -39.5) | 3531.8 (-14996.4 to 22060.1) |
| Recent Immigrants | -52.5 (-80.7 to -24.3) | -29.7 (-123. 7 to 64.3) | -139.2 (-184.7 to -93.8) | 2722.4 (-11923.65 to 17368.3) |
| Visible Minorities | -3.1 (-9.2 to 2.8) | 15.2 (-3.1 to 33.4) | -14.7 (-24.2 to -5.2) | -665.3 ( -4020.9 to 2690.3) |
| Persons Employed in Essential Services | 6.5 (-1.5 to 14.4) | -13.8 (-40.2 to 12.6) | -59.1 (-72.0 to -46.2) | 424.9 (-1977.6 to 2827.4) |
| Average Family Size | 1415.4 (1151. 5 to 1679.3) | -1675.9 (-2830.0 to -521.9) | 1353.9 (928.4 to 1779.4) | -544.9 (-37477.4 to 36387.6) |
| Multiple Family Households | -73.8 (-93. 8 to -53.8) | -18.0 (-94.3 to 58.3) | -168.9 (-213.0 to -124.9) | 7945.4 (-31720.5 to 47611.2) |
| Percent COVID Test Positivity | -0.06 (-0.11 to -0.02) | 0.21 (0.07 to 0.36) | 0.17 (0.10 to 0.25) | -7.0 (-42.3 to 28.4) |
| Active Living Environments Index | 39.8 (8.8 to 70.8) | 37.0 (-111.9 to 37.9) | 40.6 (-7.1 to 88.2) | 1913.0 (-7509.3 to 11335.4) |
| Index of Remoteness | 1018.5 (230.0 to 1806.9) | 42785 (2177.9 to 6379.0) | 4814.8 (3528.0 to 6101.6) | 48641.2 (-194231.2 to 291513.5) |
